# Event and Comment

**Published:** 1921-11

**Authors:** 


### DENTAL REGISTER

THE MAGAZINE OF DENTISTRY

Vol. LXXV NOVEMBER, 1921 No. 11

### EVENT AND COMMENT

**The Dental Economic Problem** The dental profession has been much disturbed during the past few years over the fact that so apparently little advancement in preventive dentistry has resulted from the study of this subject and that which has resulted from the practical endeavors of the clinicians. As a matter of fact, most dental diseases are becoming more and more seriously considered important health problems. There has developed in the minds of the medical profession, and through this means the public has been warned to demand more careful consideration of all dental pathologic tissues, and doctors are now more efficient in diagnostic surveillance and treatment than formerly. The dental profession is fully cognizant of the peoples needs and have a clear understanding of the medical bearings involved, and they desire to do their part in so important a humanitarian crisis; but it does not as yet clearly know how its part of the programme can be carried out. Dr. Frieselle in his presidental address says: "We need badly right now at least 1,500 more dentists, and at the same time there is an urgent demand for more efficient service." All of this the profession has known for some time, but as yet no important corrective effort has been made, unless it may be of that radical propaganda of extracting teeth, which probably is a more or less questionable means of curing disease. We have instituted prophylaxis and childrens hygiene with some hope of avoiding the serious possibilities incidental to the developmental periods of the young. But destructive peridental diseases are as much in evidence as ever before.

It is now suggested, in view of these facts, that more operative service is demanded, and that we should open the college doors more widely and force students to enter more readily by shorter and less exacting courses of study. This implies a less standard of efficiency. We are reluctant to take any such action, as this undoubtedly would discredit us if not really demoralize our ideals of professional policy and educational development. Rather than attempt to care for this situation in this way it would seem to be wiser to leave the courses of study as at present, and then try to revise our courses of study so that more emphasis can be given to the practical subjects and then eliminate all except the fundamental and essential theoretical studies. This would mean a reversion to former methods of stressing the technical arts to the detriment of cultural attainments. It will at best be several years before we can expect to get dental instruction on a par with present medical standards; and at first glance it would seem to be a mistake to take any backward moves at this time. The reasons for this advancement may be amply justifiable, but at the same time because of our inability to meet the present necessities of practice they should be considered carefully before acting.

Another suggestion has been made that may relieve the situation temporarily at least. Dr. Banzhaf of Marquette University suggests that the dentists are losing much time by failing to co-operate in conducting their methods of practice. There is too much lost motion, so to speak, and too little inclination for dentists to co-operate in caring for the public. The usual method of practice is confined to the individual dentist rather than to combining two or more dentists to work in association so as to permit each one to select the lines of practice each can do most readily. In this way less complex and burdensome business relations would be encountered and a larger output for all concerned would be secured. This method would disrupt former relations of individual responsibility and sever many intimate personal relations that all practitioners value so much. It is a fact, however, that many of the best modern practitioners are drifting into the practice of specialties; and these specialists are coming into more intimate business relations, or associations, and they find such combinations profitable and congenial. It would seem that a properly organized association of competent specialists might by conferences and closer co-operation greatly enlarge their efficiencies. Some such combination would tend to increase the capacity as well as stimulate the character of the service that could be rendered.

It would take time to change former methods, and it would not be practicable for all to combine under any practicable organization, but what is being done successfully in many places at the present time could probably be better done if a worthy cause could best be served in some such manner. Such a scheme would also make it practicable to utilize a vast number of helpful workers who could be trained as assistants in the technical fields and who could not properly serve as professional or highly-trained experts. In this way a higher grade of expert ministrations would relieve the present increased demands for educated, professionally-trained dentists. While it is true that we have now an emergency call to answer, it is not easy to see just how we can best respond without seriously endangering some of our most cherished traditions. At any rate the suggestions made, looking to a better educated profession and a more efficient business scheme, are worthy of profound and the most thoughtful considerations. Undoubtedly we shall need more dentists if they continue to serve as at present, or failing, we shall be rightfully censured for gross lack of duty, and it may be that we shall be charged with a malicious disregard for the lack of the highest interests of humanity. We don't want to be censured for incapability, nor do we propose to abandon all the good traditions our profession has evolved through ages of painstaking endeavors. We have a noble heritage as a foundation upon which we shall rear in the years to come a better science and art for the successful practice of a more noble profession than we now realize. Let us therefore continue the good work, thoughtfully, but wisely and patiently, for we may sooner than we think develop some new light that will make our way clearer and more promising.

---

**The National Dental Association** The annual meeting in Milwaukee was the largest ever attended, about 5,000 dentists attending. The technical and scientific programmes were unusually interesting and of much value. Not so much scientific research was reported, but there seems to be a constant adjustment of the artistic to the findings of the scientific results. This augurs well for future stabilization in professional practice. There was great activity in the several technical advances which were evident in the large number of those who attended the clinics. The results of the reports and papers submitted will necessarily have to await publication before even those who attended the meeting will be able to digest and to profit by them.

The educational situation was discussed at considerable length, but with no definite action that promises near adjustment to a universal standard of permanent adoption. This seems to be one of those perennial topics for which everybody seems to have a solution, but for which no scheme as yet found gives a satisfactory answer. Let us hope and work on, for as someone has said: "*There is a good time coming, boys.*"

There was the usual interest manifested in the subject of focal infections and surgical extraction of infected teeth. The subject of general prophylaxis and oral hygiene, especially for school children, was given considerable discussion and attention.

The next meeting will be held in Los Angeles, at some suitable time next summer. Dr. J. P. Buckley, formerly of Chicago, but now living in Los Angeles, was elected president.

This will probably not bring out so large an attendance, but it ought to be a delightful place to meet. There is no reason why the meeting should not induce many to attend because of the location, in face of the great distance and the disagreeable weather one meets in a long journey at such a season.

---

**An Appeal to the Dental Profession** The unsteady hand or dim eye may interfere somewhat with the physician or lawyer, in the practice of their callings, but it does not incapacitate them altogether; not so the dentist. If his health is in any way impaired, his income stops entirely, and unless he has managed to save something, he must either starve or depend upon the generosity of friends.

Owing therefore to this condition peculiar to our profession, it was found necessary to arrange some system of aid or relief whereby our unfortunate brothers who from sickness or accident are without means of support, may be helped, so that in part at least their suffering should be relieved and their burdens lightened.

It is for this purpose that the National Dental Relief was organized. The method for raising the funds is through voluntary contributions, and sale of Christmas Seals. For several years the National Secretary has been mailing to all members of the N. D. A. one hundred seals with the request $1.00 in cash be returned. However, in order to put this work on a sound basis, so that once started it should be continued without interruption, it was thought best to wait until a reserve was built up from which an income could be obtained, before extending any help.

We had hoped that the reserve fund would be collected in a short time, because we believed that no one would refuse his mite to the relief of his indigent brother.

However, we are sorry to say that this has not been the fact, considering the cause and the small amount asked, the response has not been nearly as general as we expected.

We refuse to believe that the failure to contribute is due to lack of sympathy, rather we are inclined to attribute it to negligence.

We are appealing therefore, to call your attention to a duty. Make sure that this year you do not forget our needy and dependent brothers. Also appoint yourself a committee of one and see that your neighbors likewise do not forget.

If every member will send in his dollar we will reach the desired amount with which we can begin good work. There are many deserving cases that need assistance whom we should help. Fraternally yours, THE N. D. A. RELIEF FUND COMMITTEE, Per E. S. Gaylord, *Chairman.*

### BANQUET TO C. N. JOHNSON

You dear friends have said so many complimentary and delightful things to me that I feel much embarrassed. I don't like to talk about myself, but you have left me no alternative. By the things you have done and the things you have said you have constituted me the main guy here. (Laughter.) I suppose I am always thinking on occasions of this kind, about the young men. It is good for you that the time is limited, because I have something of a message for the young men that I shall not be able to deliver.

I want to say just this word, however, to the young men that are coming up. I want to promise them that there is a reward always for conscientious effort. It may come in one way, or it may come in another, but I am going to recite just one instance, if I may, to show you what I believe to be the greatest reward that ever comes to a professional man.

Some time ago, a little after twelve o'clock at night, I was called on the phone at my home, and the statement was made that there was a woman suffering agony with a tooth about five miles from where I lived. It so happened that I had not yet gone to bed and they asked if I could not possibly come and see if I could relieve that suffering. They sent a car for me and I went, and when I stepped into that house I found a man pacing back and forth through the hall in his bathrobe almost out of his mind on account of the suffering of his wife. When I went into the bedroom I saw that woman sitting up in bed with agony pictured on her face, and her first words were: "Dr. Johnson, I think I shall lose my mind." No matter what I did for that woman, it was not fifteen minutes before she began to relax. Within twenty minutes her eyelids drooped and in less than a half-hour she was sound asleep and I was able to leave her. Ladies and gentlemen, the change of expression on that dear woman's face was a greater reward to me than all the money that could be piled in this room. (Applause.) That is the kind of reward that we get in our professional life.

One other instance, if you will permit me, I want to picture for the boys that are coming along. The older practitioners know these cases, and understand what I mean, because they have had similar experiences. Years ago there was a dear old poor couple who used to come to me as patients from the northwest side. They were loyal, genuine, good people. The old lady came with a shawl around her. They were not cultured, but they were genuine. They began to be feeble, and I suggested after a while that they would better seek a dentist in their own neighborhood because I was fearful that some injury would come to them in the traffic down in the loop, and finally I did prevail upon them to get a dentist in their neighborhood. It was only with the promise that if they got into serious trouble I would take care of them. Of course I promised. One day they came into the office; the old man was lame, it was labor for him to move at all, but they came into the office with a story that the old lady was suffering terribly and had suffered for days with a tooth, and she wondered it if was not possible that I could give her relief. I seated her in the chair and I won't tell you what I did except this, that I relieved simply a traumatic occlusion. The dentists who had been caring for her put medicine on the gum and medicine on the gum and of course it did not do any good. That tooth was raised and she said: "Doctor, I can't close my mouth." I said: "Well, I think I will relieve the pressure on that a little bit and it will be all right." "No, you can't touch the tooth," she said. But you know I could touch it and I ground it down and then I said: "Close your mouth," and she tried it, hesitatingly for just a moment, and all at once she opened and closed her mouth with a snap. Her face lighted up and she turned to me and took my hand and placed it to her lips and said: "Dr. Johnson, you are an angel." (Laughter.) I would rather have that kind of a tribute from that dear old lady, because as a professional man I gave her relief, than I would to occupy the position of president of the United States. (Applause.)

Those are the rewards. I could mention many of them, but the time is late. I will have to content myself with simply expressing my appreciation and my thanks. I want to thank all of the dear boys. Some of them have come so far that I am overwhelmed. I want to thank my other friends here not of the profession, some of my dear friends outside of dentistry. I want to thank the committee who has worked so hard for this event. I want to thank my good friend at the right, Dean Logan. I am thankful I have not had to room with that fellow in the last few weeks. I will guarantee he has asked Mrs. Logan at three or four o'clock in the morning her opinion about something connected with this banquet. I know him. I have lived with him. I tender him my thanks, and I thank the dear ladies who have come out here to grace this occasion. I wish I could tell you all what there is in my heart. I thank those who have been so generous as to give me these beautiful presents. It will be an inspiration to me as long as I live to do better and better.

I am going to close with the recital of a little prayer, about the only prayer I ever make. It is a modification of a prayer you all know, but I seldom turn the light out and place my head upon the pillow that I do not say this:

Now, I lay me down to sleep,
  I pray the Lord the faith to keep,
If I should die ere morning's sun
  I pray the Lord my work's well done.

[Dr. Johnson's replies to the toasts tendered him on the occasion of his testimonial banquet, April 11, 1921, express no finer tribute; nor more appropriately record the sincere and loyal devotion of the life he so admirably exemplifies. His closing prayer was a real benediction that might well be engraved on the memory of every loyal and devoted practitioner.—EDITOR, *Register*.]

